# A descriptive study on misidentifications of a person as a familiar person in an everyday situation

**DOI:** 10.1038/s41598-023-35094-8

**Published:** 2023-05-26

**Authors:** Yuji Itoh, Hiroshi Miura, Daisuke Shimane

**Affiliations:** 1 Department of Psychology, Keio University, Tokyo, 108-8345 Japan; 2 Faculty of Health Sciences, Kyorin University, Tokyo, 181-8611 Japan; 3 Present Address: Department of Psychology, Kyoto Women’s University, 35 Kitahiyoshi-cho, Imakumano, Higashiyama-ku, Kyoto, 605-8501 Japan; 4 Present Address: Research Center for Brain Communication, Kochi University of Technology, Kochi, 782-0003 Japan

**Keywords:** Neuroscience, Psychology

## Abstract

The purpose of this study is to show the characteristics of person misidentifications, that is, experiences in which persons are misidentified as known persons. A total of 121 participants were asked how many times they misidentified persons in the last year and details of a recent person misidentification were recorded through a traditional questionnaire. Additionally, they answered questions in a diary method questionnaire, about the details of person misidentification each time they experienced it, during the two-week survey period. The questionnaires revealed that the participants misidentified both known and unknown persons as familiar persons approximately six (traditional questionnaire) or 19 (diary method) times a year on average, regardless of whether they expected the persons to be there. They were more likely to misidentify a person as a familiar than as a less familiar person. It was also shown that the similarity of the faces of the person actually seen and the person they were mistaken for was not as high as the similarities of build and clothing. This study is expected to provide suggestions for models of person identification and enhance the research on errors.

## Introduction

One may have sometimes experienced a situation when trying to greet a friend, wherein the person approached may turn out to be a stranger and it may lead to embarrassment. The purpose of this study is to investigate how often and under what circumstances such misidentifications, that is, the misidentifications of one person as another person in everyday situations, occur. In psychology, many studies on person identification judgment have been conducted, mainly on face recognition and memory research^1,2^. For example, let us consider an experiment of witness identification of a criminal^3–6^. In such experiments, first, in the learning phase, the participants witness one person (criminal) and remember his or her appearance intentionally or unintentionally. Then, after a delay in some experiments, one or more people are shown to the participants (often presented with a facial photograph), and an identification judgment is required. The participants in the witness role pay attention to the presented person and strive to identify whether the person is the one presented in the learning phase. The cognitive activities here are quite different from those in person identification in our everyday life, where the misidentifications we focus on occur. First, we do not often judge whether a person in front of us is the same person we saw just once some time ago; rather, we more often identify a person as a specific person whom we have known for a long time. Therefore, there is usually no specific learning phase. In most cases, except in those where we are waiting for a person, we do not pay much attention to the people we see, or we are not explicitly focusing on finding a specific person. In this study, we focus on errors in the cognitive activities associated with this type of person identification.

Young, Hay, and Ellis^7^ deal with failures and difficulties in person identification. To examine a model of person identification, they collected various failures and experiences where one reported difficulties in person identification using the diary method and classified and analyzed these experiences. They collected more than 900 cases from a seven-week report of 22 participants, which included not only person misidentifications but also cases in which one was unable to recognize a known associate, one noticed a known person but could not remember who she/he was, and could not remember the full details of the person. There were more than 300 cases of person misidentification, which were the most common cases recorded. These cases were classified into those of misidentifying an unknown person as a certain known person (272 cases) and a known person as another known person (42 cases). In the former cases, visual viewing conditions were often bad, and errors were often noticed quickly, whereas in the latter, these tendencies were not as strong. The authors analyzed various types of failures and difficulties and proposed a model of functional elements of person identification similar to the model of face recognition by Hay and Young^8^. They showed the importance of building a model of person identification in which perceptual information about face, voice, build, and so on works interactively with contextual information.

Although their work had a great impact on later research, and many studies cited their work, many of these later studies focused on face recognition^9^ and memory for names^10^. There seems to be a lack of work focusing on the misidentification phenomenon itself in everyday contexts. Therefore, to address this gap, we decided to collect and analyze cases of person misidentification in everyday contexts, focusing on the phenomenon of person misidentification itself. The reasons for focusing on the misidentification among the various types of failures and difficulties are as follows: First, we often do not know much about the phenomenon of person misidentification in everyday contexts. What types of people misidentify different people, in what situations, and with what kinds of clues and regularity? Young et al.^7^ successfully worked on this issue, but this research may be insufficient, as discussed further below. Second, although some empirical studies of person identification have focused on familiar persons^11,12^, few have specifically addressed errors in mistaking a person for a familiar person. When focusing on errors, many experimental studies have used face-matching tasks^13^ or dealt with errors in judging identity with a face seen only a short time ago, as in eyewitness identification studies^14^. These studies also deal with identification and misidentification during intentional attention to the target person and intentional identification attempts; unintentional identification and misidentification have rarely been studied. In everyday contexts, however, identification and misidentification of people encountered without attention and the intention to identify them seem to occur frequently. Unintentional identification in everyday life is rarely brought to our consciousness and is difficult to study empirically, especially based on the participants’ consciousness. However, unintentional misidentification in daily life, even if it is the result of an unintentional identification attempt, may be easily recognized and can be the subject of a questionnaire survey. Therefore, it would be significant if we could clarify how often and under what circumstances a certain type of person misidentification occurs and then assert the need for research on unintentional person identification in daily life. Third, the elucidation of the phenomenon of person misidentification may have applied meanings. For example, witnesses in judicial proceedings may make statements that they have witnessed a known person at the scene of the incident. Such statements, if it is clear that the witness has no stake, may be taken at face value by investigators, judges, or jurors, but problems may arise if it is a case of misidentification. The study of person misidentification may help assess the reliability of such witness’ statements. We believe that these points simultaneously represent the significance of this research.

With these in mind, we aim to systematically develop a psychological study of person misidentification in everyday contexts, and as a first step, we conducted a study to descriptively outline the person misidentification phenomenon by reproducing and supplementing a part of the study by Young et al.^7^. We believe that the uniqueness of our research is as follows. First, we concentrate on the phenomenon of person misidentification and the collection of cases. Young et al. required participants to answer diary questionnaires each time they experienced various failures and difficulties related to person identification, as well as misidentification. This resulted in their 22 subjects reporting more than 900 cases in seven weeks, which averages out to 0.9 reports per person per day. This can be a significant load on the participants, who may have unusual concerns and attention when meeting people. To reduce the burden on the participants as much as possible and to ensure that they went about their day normally, we focused on the phenomenon of person misidentification, which was the point of interest, and collected the cases. In addition to the data collection by the diary method, we also used a questionnaire enquiring about the latest experience of person misidentification. Second, related to the first point, we included some questions in both questionnaires that Young et al. had not. These questions asked for similarities of faces and builds between a person encountered and a person misidentified. We also measured individual characteristics of cognitive failure for each participant to ascertain who is more likely to misidentify a person. In recent years, research on individual differences in face recognition and/or person identification ability has attracted attention, and various studies have been conducted^15–20^. However, what personal characteristics correlate with person misidentification in everyday life remains unclear. Therefore, we decided to take up this questionnaire that deals with various individual characteristics related to cognitive failure.

## Method

Data were collected using an ordinary questionnaire and a diary method that asked about the experience as soon as possible each time a person misidentification was experienced during the survey period.

### Participants

One hundred and twenty-one people (80 women, 40 men, one unknown) aged 19 to 84 years (*M* = 38.0, *SD* = 13.9) who were recruited using the snowball sampling method participated in the study. They were paid 3000 Japanese yen (approximately $30 USD) for their participation. Written informed consent was obtained from all the participants before the experiment. This survey was approved by the Research Ethics Committee of Faculty of Letters, Graduate School of Letters, and Graduate School of Human Relations, Keio University (no. 180230100). All methods were implemented in accordance with the relevant guidelines and regulations.

### Questionnaire

The questionnaire consisted of three parts: Pre-questionnaire, Diary, and Post-questionnaire. Pre-questionnaire, an ordinary questionnaire, was administered at the beginning of the survey period and asked about the latest experience of person misidentification before the start of the survey. Diary was a questionnaire using the diary method, and Post-questionnaire was a supplementary questionnaire asking about awareness and behavior during the survey period and at the end of the survey period. Pre-questionnaire consisted of explanations of the target experience and questions regarding the experience. The target experience was defined as one in which participants temporarily mistook a person X (known or unknown, encountered person: P_e_, hereafter) for another person Y (known directly or indirectly, person misidentified as: P_ma_), and the mistake was realized by themselves or pointed out by a third person. We provided a few examples of suitable and unsuitable experiences. The experiences excluded by the examples were those where misidentification was not sure (“Later, I just doubted that the person was my friend.”), ones where the P_e_ was not seen at all (e.g., misidentified only by voice), and ones where the P_e_ was just thought to be similar to (but not actually) a known person. A Japanese word, *hitochigai*, which appropriately refers to the person misidentification experiences this study focuses on, was used in the explanations. This was expected to exclude the reports of errors in recall of the proper names. The questions in each of the three questionnaires were as follows.

#### Pre-questionnaire

The questions were divided into three types: (1) asking about the frequency of person misidentification in the past year, (2) asking about the latest experience of misidentification, and (3) asking about everyday failure experiences. There was only one question for (1), which enquired about the frequency numerically. The questions about the latest person misidentified in (2) included questions about the P_ma_ (what kind of acquaintance: family, acquaintance, celebrity, etc.; how many times you have met the person: many times, a few times), P_e_ (how well you know the person: well, to some extent, not at all), and the situation of the misidentification (whether you were looking for the P_ma_: yes, no; to what extent you expected the P_ma_’s presence: much, to some extent, a little, not at all). Additionally, there were questions about the similarity between the faces, builds, clothing, and hair styles of the P_e_ and the P_ma_, with answers on a four-point scale ranging from 0 (not similar at all) to 3 (very similar) and a “don’t remember” option.

The questions about everyday failure experiences in (3) consisted of 24 items from Yamada’s error proneness questionnaire^21^ and two original items about person misidentification (“I misidentify a person encountered on the street as my acquaintance”; “I mistakenly greet an unfamiliar person thinking he/she is my acquaintance”). Yamada’s error proneness questionnaire contains 11 out of 25 items from the Broadbent, Cooper, FitzGerald, and Parkes^22^ Cognitive Failures Questionnaire (CFQ) and consists of three factors: action slip, cognitive narrowing, and impulsive error. Action slips are failures caused by forgetfulness or carelessness, and there are 10 items (e.g., “Do you find you forget why you went from one part of the house to the other?”), all of which are from the CFQ. There are nine items for the cognitive narrowing factor (e.g., “Do you make decisions without thinking when someone hurries you up to decide and regret it later?”), which refers to failures caused by a limited amount of information that can be processed. Impulsive errors occur because of poor action plans, and six items are included in this factor (e.g., “Do you make a promise without confirming whether the schedule for the day is free?”). One of the 25 items in Yamada’s questionnaire (“I cannot remember a person's name”) was not used because of a mistake in creating a questionnaire on the Web. Answers were required for these items on a five-point scale from 1 (not at all) to 5 (very often).

#### Diary

Diary was to be answered each time a person misidentification was experienced during the two-week survey period and included the same questions as those in (2) of Pre-questionnaire asking about the latest person misidentification. One question was added to enquire about the confidence of the misidentification with 11 steps from 0 to 100%.

#### Post-questionnaire

Post-questionnaire was to be answered once at the end of the survey period. It consisted of questions to ask, whether there were unreported misidentifications during the survey period (yes, maybe yes, no), and if so, how often (free description), and whether more attention than usual was paid to the other persons during the survey period (much more than usual, somewhat more than usual, not more than usual).

### Procedure

All surveys were conducted using email and the web. We e-mailed the persons who volunteered to participate in the survey to inform on the survey period, the experience to be reported, the estimated time to answer the questionnaire, and the reward, and asked for them to participate again. Then, we sent a supplementary explanation and links to the web pages of Pre-questionnaire and Diary to the participants. They were asked to answer Pre-questionnaire first. Each time they experienced a person misidentification within the following 14 days, they were asked to answer Diary as soon as possible. We sent the link to Post-questionnaire to the participants at the end of the survey period.

### Data analysis

Data were analyzed according to the following exploratory questions: How often do people make person misidentifications? What kinds of people are likely to misidentify other people? What are the characteristics of P_ma_s (in terms of familiarity)? What are the characteristics of P_e_s (in terms of familiarity)? In what situations are people likely to misidentify other people? What is the relationship between these characteristics (the similarity between P_e_ and P_ma_ and others)?

Before the analysis, the Shapiro–Wilk tests were conducted on the numerical data obtained in this study and revealed that most of the data were not normally distributed (normality was not rejected only for cognitive narrowing scores in the error proneness questionnaire (*p* = .157), all other *p*s < . 001). Therefore, nonparametric methods were used for the analysis of the numerical data.

## Results

All 121 participants responded to Pre- and Post-questionnaire, whereas 62 responded to Diary. First, regarding Post-questionnaire enquiring about the behavior and awareness during the survey period, 7 answered that they had failed to report and 8, that they might have failed to report misidentification, but 106 answered that they had never failed to do so. All seven who answered that they failed to report misidentification reported that they had failed to do so only once. From these results, the rate of misidentification by participants and the number of misidentifications were considered to be slightly underestimated, but generally accurate. To the question on whether they had paid more attention to people around them during the survey period than they normally would, 58 participants answered “No,” 54 “somewhat more” and nine “much more than usual.”

Since many questions were the same for Pre-questionnaire and Diary, in principle, both results are reported parallelly in the following sections.

### Frequency of person misidentifications

**Figure 1:**
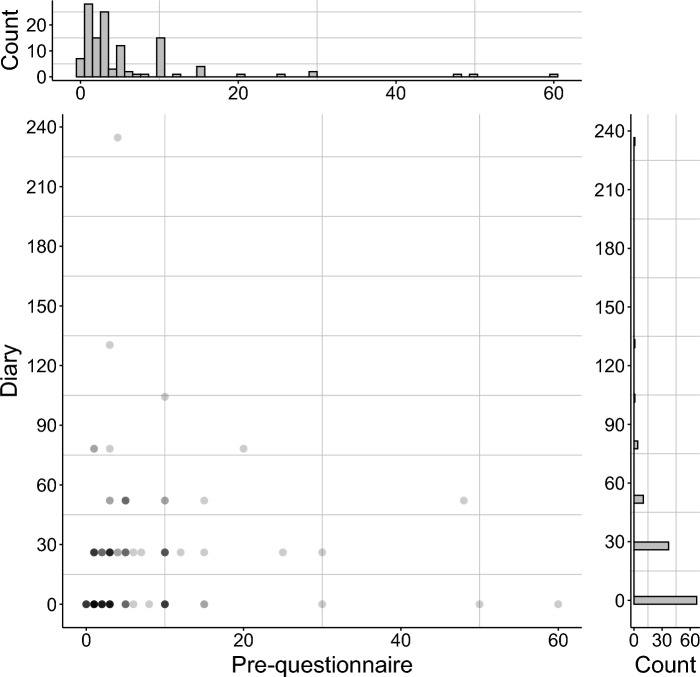
Distribution of frequencies of person misidentifications from Pre-questionnaire and Diary and their relationship. The frequency from Diary was estimated by multiplying the number of reports of misidentifications by each participant by 26.07 (365/14).

Answers to an item in Pre-questionnaire asking about the number of misidentifications during the year until the survey started, and the estimated frequencies per year from the number of reported misidentifications in Diary are shown in Fig. 1 (means were 5.93 for Pre-questionnaire and 18.75 for Diary, medians were 3 and 0, respectively). For Pre-questionnaire, among the 121 participants, 114 reported that they experienced one or more misidentifications, with a maximum of 60. The distribution was highly biased toward a smaller number of times, and most of them experienced person misidentifications once to several times a year. In Diary, although 98 person misidentification cases were reported by 62 participants, 11 were excluded from the analyses as they were from experiences outside the survey period. Eighty-seven cases from 54 participants were analyzed. Looking at the number of times converted per year (365 days), it was much larger than in Pre-questionnaire, with the average being about three times higher. A Wilcoxon signed-rank test showed that the frequency by Diary was significantly higher than that by Pre-questionnaire (*z* = 3.39, *p* < .001, *r* = .31). There was a significant positive correlation between the frequencies of misidentification in Pre-questionnaire and Diary (*ρ* = .30, *p* = .001). We compared the number of misidentifications reported by the 63 participants who paid “much” or “somewhat more” attention to people around them during the survey period with that reported by the 58 who did not pay more attention, using a Mann–Whitney U test. The former group reported more misidentifications than the latter (mean: 24.82 and 12.15, median: 1 and 0, respectively, *U* = 1449, *p* = .028, *r* = .20).

#### Gender and age differences

Regarding the question about the characteristics of the people who are likely to misidentify other people, gender and age differences in misidentification frequencies were examined. Mann–Whitney U-tests showed no gender differences in frequencies in Pre-questionnaire (mean: 6.24 and median: 3 for women; mean: 5.28 and median: 2.5 for men) and Diary (mean: 16.6, median: 0; mean: 22.2, median: 0, respectively). Regarding age differences, participants 50 years of age or over made significantly fewer misidentifications (mean: 1.81, median: 1.5) than those under 50 years of age (mean: 7.05, median: 3, *U* = 628, *p* < .001, *r* = .35) in Pre-questionnaire, while no differences were found in Diary (mean: 16.0, median: 13.0 for those 50 or over and mean: 19.5, median: 0 for those under 50).

#### Relationship with the error proneness

To see if people who are prone to making mistakes in their everyday lives are more likely to misidentify other people, we analyzed the relationship between the error proneness scores and the frequencies of misidentification. The answers to the questions about everyday failure experiences in Pre-questionnaire were averaged for each factor, and the action slip, cognitive narrowing, and impulsive failure scores for each subject were calculated. The average of the two added questions for inquiring about the experience of person misidentification was also calculated as a person misidentification score, and we obtained four error proneness scores. The Spearman’s rank correlations between these error proneness scores and the number of person misidentifications in Pre-questionnaire and Diary were then calculated (Table 1). The frequency of misidentifications per year in Pre-questionnaire showed a significant positive correlation with the action slip score of the error proneness questionnaire (*ρ* = .25, *p* = .006), and also showed a positive correlation with the person misidentification proneness score (*ρ* = .42, *p* < .001). However, the frequency of misidentifications for two weeks obtained in Diary had almost no correlation with any of these indicators. There was a significant positive correlation between the frequencies of misidentification in Pre-questionnaire and Diary (*ρ* = .30, *p* = .001).

**Table 1:** Spearman’s rank correlation between each error proneness score and frequency of person misidentification.

Error proneness score	Frequency of person misidentification	
	Pre-questionnaire	Diary
Action slip score	.25**	.05
Cognitive narrowing score	.19*	− .06
Impulsive error score	.23*	− .02
Person misidentification score	.42**	.11

The error proneness scores consisted of a person misidentification score (2 original items) and 3 measures from Yamada’s error proneness questionnaire: an action slip score (9 items), a cognitive narrowing score (9 items), an impulsive error score (6 items). Each error proneness score was calculated as the average of each item. (*p < .05, **p < .01).

### Analyses of the details of person misidentification

From here, we analyze answers on the latest person misidentifications obtained in Pre-questionnaire and those on all misidentifications in Diary. The target data were as follows: Regarding Pre-questionnaire, responses were obtained from 120 participants, excluding one who had never made any mistakes in his life. Eight more cases from eight participants were excluded from the analysis for the following reasons: Five participants reported misidentification of a P_e_ they saw on TV or movies, but because their visibility was significantly poorer than when they actually saw such a person, or the P_e_ was likely to have been dressed up for a role, it was decided not to analyze them. In addition, three participants did not understand the instructions. In total, 112 subjects were analyzed. For Diary, 87 cases from 54 participants were analyzed.

#### Familiarity of the P_ma_

What kind of persons are people likely to misidentify as? If memory representations of familiar people are more precise and easier to be identified than less familiar people, then one would misidentify someone as a less familiar person rather than a familiar one. To confirm this, Table 2(a) and (b) show the answers to the question asking what kind of acquaintance the P_ma_ was and how many times they had seen the P_ma_. It is noteworthy that in some cases, the participants misidentified people as family members, although the proportion was not very high (10.7% for Pre-questionnaire and 6.9% for Diary). As for the latter question, the exact binomial tests showed that the participants misidentified people as a well-known person whom they had seen many times significantly more often (82.1% for Pre-questionnaire and 78.2% for Diary) than as a person whom they know a little (17.9% for Pre-questionnaire and 21.8% for Diary, *p*s < .001). Misidentifying someone as a person that one knows very well, such as a family member, is not rare. People seem to tend to misidentify someone as a familiar person rather than as a less familiar person.

**Table 2:** Answer to the questions in pre-questionnaire and diary.

	Pre-questionnaire		Diary	
	Frequency	Proportion (%)	Frequency	Proportion (%)
About the person misidentified as (Pma)				
(a) What kind of known person?				
Family member	12	10.7	6	6.9
Acquaintance	78	69.6	64	73.6
Famous person	2	1.8	4	4.6
Other	20	17.9	13	14.9
(b) How often did you see the person?				
Many times	92	82.1	68	78.2
A few times	20	17.9	19	21.8
About the person encountered (Pe)				
(c) Do you know the person?				
Well	35	31.3	24	27.6
To some extent	26	23.2	23	26.4
Not at all	51	45.5	40	46.0
Concern about the person misidentified as (Pma)				
(d) Looking for the person?				
Looking for	14	12.5	16	18.4
Not looking for	98	87.5	71	81.6
(e) Expected the person to be there?				
Very much	13	11.6	14	16.1
To some extent	25	22.3	18	20.7
A little	20	17.9	8	9.2
Not at all	54	48.2	47	54.0

Frequencies totaled 112 in Pre-questionnaire and 87 in Diary.

#### Familiarity of the P_e_

Regarding the question about the characteristics of P_e_, the proportions of answers to the questions about the familiarity of P_e_ are shown in Table 2(c). It is noteworthy that, unlike the results of Young et al.^7^, the participants did not misidentify an unknown P_e_ as a P_ma_ more often (45.5% for Pre-questionnaire and 46.0% for Diary) than a known P_e_ as a P_ma_ (54.5% for Pre-questionnaire and 54.0% for Diary, *p*s by the exact binomial tests were .395 and .520, respectively).

#### Similarity between P_e_ and P_ma_

The answers to the questions about the similarity between P_e_ and P_ma_ are shown in Table 3. In Pre-questionnaire, the rate of “not similar at all” and “a little similar” was high, and of “somewhat similar” or “very similar” was low for the faces. We conducted a Friedman test on the similarity scores from 0 (not similar at all) to 3 (very similar), excluding the participants who answered “do not remember” to any of the four questions (*n* = 77). A significant main effect was detected (*χ*^2^ (3) = 37.7, *p* < .001, *η*^2^ = .16). Multiple comparisons using Wilcoxon signed-rank tests with the Bonferroni correction revealed that the similarity score for the face (1.62) was significantly lower than those of the hairstyle (2.49), body build (2.38), and clothing (2.25, *p*s < .01). Additionally, the results of Diary show a similar tendency, but the degree of similarity in clothing tends to be lower than the hairstyle and body build. A Friedman test was conducted on the 67 sets of data. A significant main effect was observed (*χ*^2^ (3) = 28.9, *p* < .001, *η*^2^ = .14), and multiple comparisons revealed that the score for face (1.54) was significantly lower than those of hairstyle (2.40) and build (2.25, *p*s < .001). This suggested that face similarity may not have as large an effect as hairstyle and body build in situations where person misidentifications occur in everyday life.

**Table 3:** The ratios of responses to questions about the similarity between a P_e_, a P_ma_ and similarity scores.

Response	Pre-questionnaire				Diary			
	Face	Hair	Build	Clothes	Face	Hair	Build	Clothes
Not similar at all	.20	.51	.44	.38	.16	.01	.03	.08
A little similar	.35	.37	.46	.33	.22	.07	.10	.14
Somewhat similar	.22	.05	.04	.06	.29	.44	.42	.31
Very similar	.13	.02	.01	.05	.24	.41	.43	.34
Do not remember	.10	.05	.05	.18	.09	.07	.02	.13
Similarity score	1.63	2.49	2.38	2.28	1.54	2.40	2.25	1.99

Similarity scores were calculated as the average by setting “not similar at all” to 0, “a little similar” to 1, “somewhat similar” to 2, and “very similar” to 3. Moreover, the participants who answered “do not remember” to any of the four questions were excluded.

#### Concern about the P_ma_

Regarding the situations in which people are likely to misidentify other persons, we focused on a mental condition of the subject of misidentifications, i.e., concern about the P_ma_. Table 2(d) and (e) show the answers to the two questions about how the participants were aware of the P_ma_ when they experienced misidentifications. As for whether or not they were looking for the P_ma_, the participants who responded that they were “looking for” (12.5% for Pre-questionnaire and 18.4% for Diary) were significantly fewer than the ones who answered that they were “not looking for” them (87.5% and 81.6%, respectively, *p*s < .001; exact binomial tests). As for whether or not the P_ma_ was expected to be there, about two-thirds of the participants (66.1% for Pre-questionnaire and 63.2% for Diary) answered, “not at all” or “a little”, which was much more than the participants who answered “very much” or “to some extent (33.9% and 36.8%, respectively, *p* < .001 and *p* = .018).” These results show that person misidentifications occur without any concern for the P_ma_ in many cases, while misidentifications sometimes occur in situations where people are looking for the P_ma_ or expecting it to be there.

#### Confidence ratings

Although not listed in Pre-questionnaire, we asked how confident the participants were that the P_e_ was the P_ma_ in Diary. The average confidence was 59.9%, and the standard deviation was 23.1. The ratings were 60% or more in 62.1% of cases and 100% in 9.2% of the cases. Thus, the participants (mis)identified P_e_ as P_ma_ not in a very skeptical manner.

### Relations among the questions

Chi-square tests were conducted to examine the relationships among the answers to the five questions, i.e., the questions about the familiarity of the P_ma_ (two questions), the familiarity of the P_e_ (one question), and the concern about the P_ma_ (two questions) with α = .01. The four categories of the answers to the question on the expectation of the P_ma_’s presence were merged into two categories, “expected” (“much,” “to some extent” and “a little”) and “not expected” (“not at all”). As a result, in both Pre-questionnaire and Diary, a significant relationship was found only between the degree to which the P_ma_ was seen and the familiarity of the P_e_ (Pre-questionnaire: *χ*^2^(2) = 19.0, *p* < .001, Cramer’s *V* = .41, Diary: *χ*^2^(2) = 13.0, *p* = .001 Cramer’s *V* = .39, Table 4). Residual analysis showed that, in both Pre-questionnaire and Diary, very familiar P_e_s were easily misidentified as P_ma_s who had been seen many times (Pre-questionnaire: *p* < .05, Diary: *p* < .005), and slightly familiar P_e_s were easily misidentified as P_ma_s who had been seen a few times (Pre-questionnaire: *p* < .01, Diary: *p* < .005).

**Table 4:** Relation between the degree to which the P_ma_ has been seen and the familiarity of the P_e_.

How often have you seen the P_ma_?	(a) Pre-questionnaire			(b) Diary		
	How well do you know P_e_?			How well do you know P_e_?		
	Well	To some extent	Not at all	Well	To some extent	Not at all
Many times	33	14	45	24	13	31
A few times	2	12	6	0	10	9

The total frequencies were 112 in Pre-questionnaire and 87 in Diary.

To examine the relationship among the above five questions and the similarity scores of face, hairstyle, body build, and clothing of the P_e_ and the P_ma_, we conducted Mann–Whitney U tests on similarity scores with the answers to these five questions as independent variables (Some categories are collapsed). The following significant differences were found: α = .01. In Pre-questionnaire, facial similarity was higher when the presence of P_ma_ was not expected at all (mean: 1.96, median: 2) than when it was expected (mean: 1.42, median: 1, *U* = 868, *p* = .004, *r* = .29) and clothing similarity was lower when P_ma_ was not expected (mean: 2.02, median: 2) than when it was (mean: 2.45, median: 3, *U* = 1365.5, *p* = .008, *r* = .28). Similarly, in Diary, facial similarity was higher (mean: 1.96 vs. 1.29, median: 2 vs. 1, *U* = 488, *p* = .004, *r* = .32) and clothing similarity was lower (mean: 1.72 vs. 2.41, median: 2 vs. 3, *U* = 1019.5, *p* = .001, *r* = .38) when P_ma_ was not expected than when it was. In addition, only in Diary, hairstyle similarity was lower when P_ma_ was the person whom the participant had seen a few times (mean: 1.94, median: 2) than many times (mean: 2.45, median: 2, *U* = 749, *p* = .008, *r* = .29) and it was also lower when P_e_ was a known person (mean: 2.16, median: 2) than an unknown person (mean: 2.57, median: 3, *U* = 558.5, *p* = .007, *r* = .30).

### Comparison between the methods

In this study, we used two questionnaires to examine the frequency of person misidentification and the characteristics of each misidentification, i.e., a conventional retrospective questionnaire in Pre-questionnaire and a diary method questionnaire in Diary. As we already reported the differences regarding the frequency of misidentification, here, we describe a comparative analysis of the results regarding the characteristics of misidentification.

First, we conducted chi-square tests to see if the patterns of the answers were different between the two questionnaires and found no significant differences (for the expectation of the presence of P_ma_, *χ*^2^(2) = 6.45, *p* = .09, Cramer’s *V* = .18, all other *p*s > .24, Cramer’s *V*s < .09). Then, the similarities between P_e_ and P_ma_ were also compared with Mann–Whitney U tests and no significant differences were detected (*p*s > .19, *r*s < .11). As for the characteristics of misidentification, the results of the two methods seem to agree well.

## Discussion

### Frequency of person misidentifications

#### Estimation of the frequency

In this study, we investigated person misidentification, in which people mistakenly identify a witnessed person as a known person. We used an ordinary questionnaire and a diary method to determine how often and what kinds of people misidentified other people. We found that most of the participants had experienced person misidentification at least once a year, and the frequency was considered to be 6 (the participants’ estimates in Pre-questionnaire) to 19 (estimated from the numbers of reports in Diary) per year on average. There were significant differences between the results from the two methods, and how should we estimate the true frequency? The retrospective question on the frequency per year in Pre-questionnaire was dependent on memory and was likely to underestimate the frequency if it was difficult to remember the misidentification cases. In contrast, in Diary, overestimation may have occurred given that the participants who answered in Post-questionnaire that they were more aware of the people they encountered than usual reported a higher frequency than those who answered they were not. Thus, it can be inferred that the frequency of the misidentification lies between the two values, but further studies are needed to verify this or to determine where in between. The frequency of those who answered that they had not paid more attention to others than usual during the survey period, approximately 12 times a year, may be the best estimate for this study.

Considering what was mentioned above, there might exist a problem, that is, the requirement to report person misidentification would make the participants pay more attention than usual to the people they meet, thereby increasing misidentifications. It is possible that an experience that would not normally be recognized as misidentification could be reported as one. This is suggested by the abovementioned difference in the number of reported misidentifications between the participants who answered that they paid more attention to the person they met than usual or not. Furthermore, the fact that the participants in Young et al.^7^ reported much more person misidentifications (106 per participant per year) than ours might also suggest the possibility. Their participants had to report any cases of failure or difficulty related to person identification, and this demanding situation might have made them see an encountered unknown person as someone among their acquaintances even in a situation where they would usually pay little or no attention to the person. Of course, it is possible that their participants were more likely to notice misidentification than ours, so it is necessary to examine the frequency of misidentifications in everyday life using other methods. Another thing to note in this study is that we collected only the cases in which misidentifications were noticed, such as being pointed out by others or by oneself, so it is considered that there may be cases where misidentifications occurred but were unnoticed. Anyway, it seems that person misidentification is not a very rare phenomenon.

#### Individual differences

Another noteworthy result regarding the frequency of person misidentifications is the significant positive correlation between the frequencies from Pre-questionnaire and Diary. Recent studies on face recognition and person identification have revealed that there are individual differences in these functions among normal people and that their consistencies across tasks or situations have been found in some studies and have not in others^15–20^. The results of the present study suggest that there are consistent individual differences in the likelihood of person misidentification among people in everyday situations, despite the problems of memory in retrospective methods and instability of data due to the short duration of the diary method, as discussed later. Confirming the consistency of this individual difference and examining the relationship with other individual differences in face recognition and person identification would be an important topic for future research.

#### Gender and age differences

Regarding the types of participants that were more likely to misidentify persons, the results were not consistent. Regarding the gender difference, we observed no difference in both Pre-questionnaire and Diary. However, as for the age difference, older participants (50 years of age or older) estimated smaller number of misidentifications than younger ones (under 50) in Pre-questionnaire whereas there was no difference between the numbers of reported misidentifications in Diary. This is similar to the phenomenon called age prospective memory paradox where older participants surpass younger ones or there are no differences in the performance of naturalistic prospective memory tasks, whereas younger ones surpass older ones in laboratory tasks^23–25^. However, in this study, the misidentification occurred in a naturalistic situation in both questionnaires, and the difference in the results cannot be attributed to the tasks. A possible explanation might be that answering Pre-questionnaire relied on memory, and that decrease in access to memory for individual misidentification events in older age led to lower estimates, or that although the increase in meta-cognitive skills and the decline in daily activities decrease misidentifications in older people, the age difference was not detected in Diary due to the short survey period, etc. We believe that this is a topic for future study.

#### Error proneness and the frequency

There was another inconsistency between the results of Pre-questionnaire and Diary. It was shown that those who had a high tendency to make action slips and impulsive errors, as measured by the error proneness questionnaire, and those who thought that they often misidentified persons were more likely to misidentify persons. However, these were seen only for the frequency of misidentifications obtained by Pre-questionnaire, and data from Diary did not show these relationships at all. This discrepancy may arise from the fact that the frequency of misidentifications in Pre-questionnaire reflects the subjective perception of one's tendency to make errors or to fail, but does not reflect the frequency of actual misidentifications, or consideration of the frequency of person misidentifications influencing the answers to the questions in the error proneness questionnaire. In these cases, the relationship between error proneness and the frequency of misidentifications is spurious. Alternatively, like age difference, it is possible that the two-week period of the diary survey was not long enough to reflect individual characteristics because the occurrence of misidentifications was more dependent on the environmental factors at that time. In the present study, only Pre-questionnaire showed that people with a self-perception of being more prone to person misidentification were more likely to actually misidentified people. In recent years, research has been conducted on the relationship between metacognitive evaluations of one's face recognition ability and actual face recognition ability, with mixed results^26–28^. If we can clearly demonstrate the presence or absence of the relationship shown by only one of the methods, it would provide new insight into the debate about the accuracy of this metacognition. Further data should be collected and considered with regard to these various points, and conclusions on this relationship should be withheld.

### Situations, person encountered, and person misidentified as

Regarding the situation wherein person misidentification is likely to occur, only limited aspects have been examined, that is, whether misidentification is more likely to occur in situations where P_ma_ is likely to be present. We found that misidentification does not occur only when the P_ma_ is being looked for or when the P_ma_ is expected to be there but it is more likely to occur when P_ma_ is being looked for or expected to be there. Regarding the P_ma_, people are sometimes misidentified as very familiar, instead of somewhat familiar, acquaintances. Although the proportion is not so large, it has been shown that someone may be misidentified as a very familiar person, such as a family member.

As for P_e_, misidentification occurred regardless of whether an unknown person or a known person was seen, and in both the ordinary and diary questionnaires, the majority were misidentifications wherein a known person was mistaken for another known person. Additionally, Young et al.^7^ reported both types of misidentifications, but the overwhelming majority were cases of misidentifying unknown persons (272 out of 314 cases), which is different from our result. The reason for this is unclear, but it is possible that the participants in Young et al.'s study were more aware of the unknown people they encountered because they were required to report a wider range of experiences of failures and difficulties related to person identification. In addition, since many of our participants were housewives and office workers versus the students in Young et al., it is possible that the latter had more opportunities to meet unknown people than ours.

### A model of person (mis)identification

From the analysis of the relationships between the questions, it is shown that when the P_e_ is a known person but familiarity is low, the person tends to be misidentified as a person with low familiarity who the participant has met only a few times, whereas when the P_e_ is a highly familiar person, the person tends to be misidentified as a highly familiar person. This may be explained by a person identification model, such as Young et al.^7^ or the interactive activation and competition model of face recognition by Burton, Bruce, and Johnston^29–31^. In this model, let us consider that P_e_ is identified as a P_ma_ when the activation level of P_ma_’s person identity node (PIN) reaches a threshold. A person’s PIN and units like a face recognition unit (FRU) or a voice recognition unit (VRU), which receives perceptual input of the face, body build, hairstyle, voice, and so on, activate mutually. A PIN and semantic information units (SIUs) that encode personal information, such as occupations or residential areas, also activate mutually. When one encounters a P_e_, perceptual inputs are received from them, and when several PINs are activated by the perceptual inputs, the individuals associated with these PINs then become candidates for the P_e_’s identity. Among the candidates, one whose PIN activation reaches the threshold first wins the competition and is identified. A person with high familiarity tends to be a strong candidate for person identification judgment even when the person is not actually encountered because the links connecting the P_e_’s perceptual features and the PIN through units, such as the FRU or VRU, and/or the links connecting the PIN and SIUs are strong. Thus, the probability for the person to win the competition is relatively high, even if the familiarity of the P_e_ who is actually encountered is high. A person with low familiarity cannot become a strong candidate easily and cannot win the competition when a strong candidate with high familiarity exists. However, if P_e_ is low in familiarity and not a strong candidate, even a person with low familiarity can win the competition and be misidentified. Another possibility is that people use familiarity as an important clue when identifying a person, and often identify a P_e_ on this basis.

With regard to models of person misidentification, modeling by erroneous activation of PINs that are not modality-specific appears plausible, as suggested by Young et al.^7^. On the other hand, in a recent EEG study, Wirth et al.^13^ showed that person misidentification is caused by erroneous activation of visual memory representations of faces and not by higher-order decision processes. This would indicate that in the above model, the misidentification is due to erroneous activation of the FRUs, not the PINs. However, this study was based on a face-matching task that involved making an identity judgment with an unfamiliar face seen immediately before, and it is possible that a different mechanism is at work for the identification of a known person. Some recent neuropsychological studies have also argued that the activation of modality-specific units rather than modality-unspecific units like PINs is involved in person identification^12,32^. It would be necessary to conduct empirical research from the perspective of whether modality-specific or modality-unspecific units are involved in misidentification in everyday situations.

### Similarity between the P_e_ and the P_ma_

We asked about the similarity of the face, hairstyle, body build, and clothing of the P_e_ and P_ma_, and found that the similarity of the face was rated lower than the others, and that the similarity of clothing was rather low. This similarity is based only on memory, and it is unclear whether it reflects an objective similarity. It is also unclear whether they recalled the appearance of each person and evaluated their similarity, or whether they were based on the memory of the impression of similarity. It may reflect a memory such as “Their faces were not very similar, but I made a mistake because their build was similar.” Nevertheless, the face as a cue in everyday person identification seems to have a relatively small weight. These results seem inconsistent with those of Young et al.^7^. They did not ask for similarities between P_e_ and P_ma_, but for the most important sources of identification failure. According to their results, the face was chosen at a high rate of approximately 30%, along with the hairstyle, and it was significantly higher than the build and clothing. If the misidentifications are caused by similarity of the sources, one would expect a high degree of similarity of the faces that were given the most weight. The reason for this inconsistency might be that Young et al.'s participants paid more attention to the people they encountered than ours because of the task demand and looked closely at the faces of the persons they met.

Another possibility is that people may subjectively think that they have judged by face, but actually judge by other sources, such as body built. Researchers have examined the extent to which people use information from the face, body, and other sources in person recognition decisions and have shown that people rely strongly on the face, even though information from the body is also useful. For example, Burton et al. showed that in recognition of familiar persons, people obtain some information from the body and gait, but they also rely on the face, even when they are recognizing with poor quality video images^33^. On the other hand, O'Toole et al. showed that in same-different judgments of unfamiliar persons in videos and still images, the performance was better for the video presentation when the body was included, and that when persons were presented in video, attention was paid to the body as much as to the face^34^. Furthermore, Rice et al.^35^ showed that body information is as useful as face information in the same-different judgments of unfamiliar persons under conditions in which the faces are similar to each other and judgments based on faces are difficult to make. Considering these findings, it seems likely that in the everyday situation, people misidentify a person as another person based on information from the body and other sources with little attention to the person’s face while assuming it is based on the face.

As a related result of the above discussion, in both the ordinary questionnaire and the diary method, the higher the degree of expectation about the presence of the P_ma_, the lower the facial similarity and the higher the clothing similarity. These results could be explained by the view that person identification does not depend only on the degree of congruence between the characteristics of a specific person’s memory and those of the person actually seen (degree of PIN activation by perceptual input^7,36^), but on the function of the cognitive system, such as expectations and contextual judgments. When the cognitive system suggests that it is a specific person, visual cues such as clothing can be used even in bad visual conditions and lead to quick decisions. This is true even if there exists a high possibility of making a mistake. In cases where careful judgment is required, dependence on the face is considered high.

### Difference between the methods

In this research, the same participants were asked about the frequency of person misidentifications in the previous year and about the latest misidentification using an ordinary questionnaire, and about misidentifications experienced in the two weeks of using the diary method. Many of the questions used in the two methods were identical. Therefore, the question arises: Which is the more appropriate method to capture the person misidentification phenomenon? For an ordinary questionnaire that relies on participants’ memory more than a diary method, it is possible that results tend to be influenced by forgetting or erroneous recall more in the former method than in the latter. There is a possibility that the participants’ folk theory on the psychological phenomenon would have a greater influence on the ordinary questionnaire. In these respects, a diary method would be advantageous. However, the pressure to report person misidentification in the diary method may increase attention to people encountered in everyday life, which may affect the process of person identification or misidentification. It is also difficult to use many questions in the diary method because participants have to answer the questions during their daily activities. This suggests the superiority of an ordinary questionnaire.

When the results of the two methods are compared, there were discrepancies in the responses to some questions, such as the frequencies of person misidentifications and the relationship between the frequencies and personal characteristics regarding error proneness. However, most of the results were similar. This indicates that many of the results of this study are reliable. This might also indicate that the degree of participants’ attention to people encountered did not have large effects on many aspects of person misidentification, and the details of the impressive misidentification experience that the participants reported were relatively well remembered. Thus far, both methods seem to have been valid for approaching most aspects of the person misidentification phenomenon. However, because some discrepancies were observed in the results, it seems better to use both methods together in future research to complement the defects of each.

### Limitations and future directions

This research is exploratory, and the questions and research periods may not be sufficient. Although person misidentifications are not very rare, a period of two weeks was not sufficient. In fact, in this research, 54 out of 121 participants experienced and reported one or more misidentifications during the two-week period of data collection by the diary method, but the period should be long enough for most of the participants to make one or more misidentifications. In addition, a larger sample size will be required to analyze the relationship among the questions. There were some questions that could not be analyzed in this research, such as those for free description of the situation; thus, it is necessary to devise them.

However, even if these improvements are made, there are limitations to this research. Similar to the study by Young et al.^7^, this study can only be applied to cases in which misidentification is noticed, such as by oneself or by being pointed out by others. Besides these cases, there are cases of unnoticed misidentification, but these cannot be targeted. Approaching these cases is important in understanding the person misidentification phenomenon as well as in the application of the reliability of a testimony that a witness saw a known person. In addition, beyond these noticed or unnoticed misidentifications, there are a significant number of cases where the encountered known person was correctly identified or an encountered person was not judged to be a known person (including correct rejections and misses). These are also extremely difficult to grasp using questionnaires, but they are very important for studying person identification and misidentification in everyday life. Although it may be difficult to intentionally induce the kind of person misidentifications we studied in this research, we believe that conducting experimental studies both inside and outside the laboratory is also necessary to concurrently examine person misidentifications and other correct and erroneous person identifications to comprehensively understand this phenomenon.

Finally, in further developing the research, it is also important to consider aspects related to its application. For example, a testimony of witnessing an acquaintance committing a crime at the scene is generally strongly relied upon, but its reliability has not been well studied empirically^37^. Errors in such situations are precisely the kind of misidentifications we have focused on in this study, and while this study suggests that they do exist, it does not suggest anything about the rate at which they occur. Another important future direction would be to relate psychological research on person misidentification to computer vision research, which has recently made great progress in the areas of face recognition and person identification^38^. Especially for tasks that require judgments about multiple persons in the field of vision as to whether they are one of the many persons in the database, we would expect a reciprocal impact when a psychological study of misidentification is developed.

## Summary and conclusion

In this research, we examined the person misidentification phenomenon in everyday situations using ordinary and diary method questionnaires. The results revealed that participants experienced more than a few misidentifications per year on average. In a previous study by Young et al.^7^, participants misidentified a known person as another known person and also misidentified an unknown person as a known person. However, contrary to the results of Young et al., it was shown that there were more misidentifications of the former type than of the latter. We also found that participants sometimes misidentified a person as a very familiar person.

We found that the similarity of the faces of P_e_ and P_ma_ was not as high as that of the body build and hairstyle. Person misidentifications occurred even when the participant was unaware of the P_ma_. In cases when one was aware of the P_ma_, that is, where one was looking for the P_ma_ or expected that the P_ma_ was there, the similarity of the faces of the P_e_ and P_ma_ was rated lower than when one was not aware of the same.

Regarding a specific part of failures and difficulties in person identification that Young et al.^7^ targeted, we believe that our study can capture the person misidentification phenomenon in more natural circumstances, where participants did not pay much attention to the people around them. The differences between the results from Young et al. and ours, and between the results from the ordinary questionnaire and the diary method in our research have highlighted the importance of the methodological issues in this kind of research.

Although there are few empirical studies on person misidentification in everyday situations, it is important to research this phenomenon and we hope that many empirical studies will be conducted, both inside and outside Japan. As the phenomenon of person misidentification must be very socially and culturally determined, it is not clear as to what extent our data can be generalized. However, this research is significant as a reference for future cross-cultural comparisons and for further questionnaire and experimental studies in Japan.

## Data Availability

The datasets generated during and/or analyzed during this study are available from the corresponding author upon request.
